# Revisiting the role of IL-27 in obesity-related metabolic diseases: safeguard or perturbation?

**DOI:** 10.3389/fimmu.2024.1498288

**Published:** 2025-01-21

**Authors:** Jinyang An, Donghua Fu, Ximei Chen, Conghui Guan, Lingling Li, Jia Bai, Haihong Lv

**Affiliations:** ^1^ The First Clinical Medical College of Lanzhou University, Lanzhou, Gansu, China; ^2^ Department of Endocrinology, The People’s Hospital of Yuzhong County, Lanzhou, Gansu, China; ^3^ Department of General Medicine, Zhengzhou Yihe Hospital affiliated to Henan University, Zhengzhou, Henan, China; ^4^ Department of Endocrinology, The First Hospital of Lanzhou University, Lanzhou, Gansu, China

**Keywords:** il-27, obesity, metabolic diseases, cardiovascular diseases, abnormal glucose metabolism, lipid metabolism disorder, immune inflammation, a new target

## Abstract

The prevalence of metabolic diseases, such as obesity, has been steadily increasing in recent years, posing a significant threat to public health. Therefore, early identification and intervention play a crucial role. With the deepening understanding of the etiology of metabolic diseases, novel therapeutic targets are emerging for the treatment of obesity, lipid metabolism disorders, cardiovascular and cerebrovascular diseases, glucose metabolism disorders, and other related metabolic conditions. IL-27, as a multi-potent cytokine, holds great promise as a potential candidate target in this regard. This article provides a comprehensive review of the latest findings on IL-27 expression and signal transduction in the regulation of immune inflammatory cells, as well as its implications in obesity and other related metabolic diseases. Furthermore, it explores the potential of IL-27 as a novel therapeutic target for the treatment of obesity and metabolic disorders. Finally, an overview is presented on both the opportunities and challenges associated with targeting IL-27 for therapeutic interventions.

## 1 Introduction

### 1.1 The growing global burden of obesity and its consequences

Obesity has become one of the most pressing global public health challenges. According to the World Health Organization, over 1 billion people worldwide are currently classified as obese, and this number continues to rise (1). Obesity is not merely an isolated health issue; it is strongly associated with several serious metabolic disorders, including dyslipidemia, abnormalities in glucose metabolism, cardiovascular diseases, and gastrointestinal disorders. Particularly in cases of abdominal obesity, lipid metabolism dysfunction is frequently observed, contributing to an increased risk of atherosclerosis and cardiovascular diseases (2). When hyperglycemia accompanies obesity and its related metabolic disorders, the risk and progression of complications are significantly amplified (3). Over the past two decades, mortality from metabolic diseases has steadily increased, with obesity accounting for approximately 40% of these deaths, contributing to 5 million fatalities in 2019 alone (4). These conditions not only severely impact patients’ quality of life but also place a significant burden on healthcare systems. Given the severe consequences of obesity and its related metabolic diseases, extensive efforts have been made to investigate its underlying causes and to develop effective treatment strategies and therapeutic interventions.

### 1.2 Hypotheses underpinning the complex etiology of obesity

Researchers acknowledge that obesity is a complex disease influenced by multiple factors, each supported by distinct hypotheses. The energy balance hypothesis posits that obesity arises from a prolonged imbalance between energy intake and expenditure, with excessive calorie consumption and insufficient physical activity leading to fat accumulation (5). The genetic hypothesis highlights the critical role of genetic predisposition in obesity, with genome-wide association studies identifying genes such as FTO and MC4R, which are involved in appetite regulation, energy metabolism, and fat distribution (6). Additionally, the dietary habits hypothesis emphasizes the impact of poor dietary patterns, such as a high intake of ultra-processed foods and sugary beverages, coupled with low fiber consumption, which contribute to excessive calorie intake and nutrient imbalance (7). Moreover, the environmental factors hypothesis points to lifestyle changes, including urbanization, limited access to healthy foods, and reduced physical activity, as factors creating obesogenic environments (8). Lastly, the microbiome hypothesis highlights the gut microbiota’s role in energy harvest, lipid metabolism, and systemic inflammation, where dysbiosis—or an imbalance in microbial communities—is linked to obesity (9). These interconnected hypotheses underline the multifactorial nature of obesity and its complex etiology.

At the molecular level, obesity involves several intertwined mechanisms. The insulin resistance hypothesis proposes that impaired glucose uptake by cells leads to hyperglycemia, increased lipogenesis, and fat deposition (10). The leptin resistance hypothesis suggests that in obese individuals, leptin, a hormone regulating appetite and energy expenditure, becomes ineffective, disrupting satiety signals and promoting overeating (11). The adiponectin hypothesis posits that reduced levels of adiponectin, an adipokine enhancing insulin sensitivity and anti-inflammatory responses, contribute to metabolic dysfunction (12). Furthermore, the brown adipose tissue (BAT) function hypothesis focuses on thermogenesis, where reduced BAT activity or dysfunction decreases energy expenditure and favors fat accumulation (13). Finally, the microbiome-metabolism interaction hypothesis explores how gut microbiota regulate nutrient absorption, energy storage, and inflammatory responses, with dysregulated interactions potentially driving obesity-related metabolic complications (14). These molecular mechanisms provide a deeper understanding of obesity’s pathophysiology, bridging biological processes with the broader hypotheses.

### 1.3 Obesity-associated metabolic disorders and their pathophysiology

Obesity and its related metabolic disorders, including dyslipidemia (15), type 1 diabetes (T1DM) (16), type 2 diabetes (T2DM) (17), cardiovascular disease (18), cerebrovascular disease (19), hypertension (20), and gut health dysregulation (21), pose significant public health challenges worldwide. Dyslipidemia, a common complication of obesity, is characterized by elevated triglycerides, low high-density lipoprotein cholesterol (HDL-C), and increased small, dense low-density lipoprotein particles (LDL-C), which collectively accelerate the development of atherosclerosis (22). For instance, studies have shown that obesity significantly increases the risk of heart disease and stroke. For every 5 kg/m² increase in BMI, overall mortality rises by 30% (23). Additionally, insulin resistance, a hallmark of obesity, exacerbates glucose metabolism abnormalities, further contributing to cardiovascular risk (24).

When obesity and related metabolic diseases are accompanied by hyperglycemia, the risk of complications such as diabetic kidney disease (DKD), retinopathy, and cardiovascular diseases increases significantly. Chronic hyperglycemia accelerates the progression of these complications by promoting advanced glycation end-product (AGE) formation, oxidative stress, and endothelial dysfunction (25). For instance, studies have shown that individuals with obesity and diabetes are twice as likely to develop DKD and face a 2- to 4-fold higher risk of cardiovascular events compared to those without these conditions (26).

### 1.4 Inflammation as a central mechanism and potential therapeutic targets

Chronic low-grade inflammation, driven by the activation of immune cells and overproduction of pro-inflammatory cytokines, serves as a common hallmark of these conditions (27). In obese individuals, adipose tissue undergoes pathological remodeling, including the infiltration of pro-inflammatory immune cells such as macrophages and T cells (28). These immune cells secrete key inflammatory cytokines, such as interleukin-6 (IL-6) (29), tumor necrosis factor-alpha (TNF-α) (30), and interleukin-1 beta (IL-1β) (31). IL-6 disrupts insulin signaling by increasing the phosphorylation of insulin receptor substrates, thereby promoting insulin resistance (32). TNF-α exacerbates lipolysis in adipocytes, leading to the release of free fatty acids and further aggravating lipid accumulation and systemic inflammation (33). Similarly, IL-1β enhances the recruitment of immune cells to adipose tissue, perpetuating a vicious cycle of inflammation (34). Collectively, these inflammatory markers disrupt metabolic homeostasis, forming the pathological basis for complications such as type 2 diabetes, atherosclerosis, and cardiovascular diseases (35).

Given the critical role of these inflammatory markers, significant research has focused on targeting IL-6 and TNF-α to mitigate metabolic inflammation. For instance, anti-TNF-α therapies, such as infliximab, have shown some success in reducing systemic inflammation in autoimmune diseases like rheumatoid arthritis (36). However, their efficacy in addressing obesity-induced inflammation and associated metabolic dysfunctions remains limited. Studies indicate that these therapies often fail to fully restore metabolic balance because they do not address the complex interplay between pro-inflammatory and anti-inflammatory pathways in obese individuals (37). This limitation underscores the need for alternative therapeutic targets capable of more comprehensively regulating chronic inflammation.

Recent studies have highlighted IL-27 as a key cytokine involved in the regulation of immune inflammation and metabolic homeostasis (38). Unlike classical inflammatory markers, IL-27 exhibits dual regulatory properties, acting as both a pro-inflammatory and anti-inflammatory mediator depending on the context. Studies have shown that IL-27 inhibits the differentiation of Th17 cells, a major source of pro-inflammatory cytokines (39), while promoting the expansion of regulatory T cells (Tregs) to restore immune homeostasis (40). Additionally, IL-27 has been demonstrated to suppress lipid accumulation in macrophages and modulate adipose tissue inflammation, indicating its potential in resolving the chronic inflammatory state associated with obesity (38). By modulating immune responses in adipose tissue, islets, vasculature, and the gut, IL-27 has the potential to both exacerbate metabolic dysfunction and exert protective effects through its anti-inflammatory properties, thereby positioning itself as a potential therapeutic target for these diseases.

## 2 Pathogenesis, treatment and targets of obesity

Obesity is a multifactorial chronic metabolic disease influenced by an intricate interplay of genetic predispositions, environmental exposures, dietary patterns, and lifestyle behaviors (41). The World Health Organization defines obesity as an adult body mass index greater than 30 kg/m² (42). Lifestyle interventions, such as dietary restrictions and increased physical activity, are considered important methods for weight management (43). However, the effects of these interventions are generally difficult to sustain and are prone to rebound (44, 45). Obesity induces significant changes in both innate and adaptive immune systems within adipose tissue, characterized by an increased infiltration of pro-inflammatory macrophages (M1) and CD8^+^T cells, along with a reduction in anti-inflammatory Tregs and Th2 cells. These immune cell alterations create a chronic inflammatory environment that is perpetuated by feedback loops between immune cells and adipocytes, maintaining a prolonged state of low-grade inflammation and contributing to systemic metabolic dysfunctions (46), with alterations in the proportion and function of immune cells, and this inflammatory state persists for a prolonged period (47). Research indicates that cytokines secreted in the inflammatory state of adipose tissue do not decrease with subsequent weight loss (48). The inflammatory environment significantly increases the likelihood of obesity rebound, known as “obesity memory,” with the key factor being the persistent inflammatory cells in the adipose tissue (49).

In addressing the above mechanism, a small-scale population study attempted dietary interventions by providing participants with anti-inflammatory foods such as olive oil, leafy vegetables, and fruits. The study observed that an anti-inflammatory diet had some inhibitory effect on weight rebound, but this conclusion requires further verification (50). Research indicates that weight loss achieved solely through dietary changes tends to rebound due to the irreversible inflammation in adipose tissue and the hypothalamus after weight loss, with leptin resistance persisting (51). Similarly, after achieving weight loss through increased physical activity, a high level of exercise must be maintained to suppress weight rebound, and sustaining high-intensity exercise over the long term is challenging (52). In terms of pharmacological treatment, glucagon-like peptide-1 (GLP-1) receptor agonists have become a preferred option for treating obesity due to their remarkable efficacy, as they can mimic insulin secretion and control appetite. However, most individuals regain their previous weight after discontinuing these medications, and health indicators such as blood pressure, blood glucose, and cholesterol levels also worsen (53). Thus, the challenge in obesity treatment is not only to achieve successful weight loss but more importantly, to maintain the weight loss outcomes.

After immunometabolic regulation, they polarize to the pro-inflammatory M1 phenotype, producing various pro-inflammatory factors such as IL-1β, IL-6, TNFα, and iNOS, shaping an inflammatory obese environment that leads to multiple obesity-related metabolic diseases (54). Thus, targeting the regulation of inflammatory factors to reverse the inflammatory state in obese tissues, eliminate “obesity memory,” and achieve sustained weight loss might be a promising direction for future treatments. In adipose tissue, adipose-associated macrophages are most closely related to inflammation. In a study on the regulatory mechanisms of obesity-induced inflammation and metabolic disorders, researchers observed that TRIM21, an E3 ubiquitin ligase, upregulates inflammatory factors (such as TNF-α and IL-6). They revealed that targeting these key molecules of TRIM21 can alleviate chronic inflammation caused by obesity, thus improving metabolic health (55). Lipids in adipocytes upregulate interleukins, particularly IL-1α and IL-1β, causing immune cell infiltration that disrupts adipocyte function, promotes inflammation, and leads to insulin resistance, resulting in obesity-related metabolic diseases like type 2 diabetes and atherosclerosis (56). However, inflammation is a complex and interconnected process, and interleukins IL-18 and IL-33 in the interleukin family have been demonstrated to mitigate metabolic disorders and play a role in alleviating insulin resistance and obesity (57).

We can observe that despite the structural and origin similarities of interleukins and other cytokines, their roles in obesity differ significantly. Within this complex context, IL-27, as a multifunctional cytokine, has increasingly garnered attention for its role in obesity-related metabolic diseases. IL-27 not only can exert anti-inflammatory effects under specific conditions, but it may also promote weight loss during the “inflammatory state” by modulating immune cell responses, thus profoundly impacting obesity and its related metabolic disorders (38). Thus, investigating the specific role of IL-27 in obesity and its potential therapeutic value is of great importance.

## 3 IL-27 and its receptor

### 3.1 IL-27 factor profile

Interleukins were initially named for their role in leukocytes, but subsequent research has shown that they can be produced by multiple cell types and exert widespread effects throughout the body. However, the naming and classification system of interleukins continues to be refined and is still evolving (58). IL-27 is a distinctive factor within the interleukin family, playing a dual role in both anti-inflammatory and pro-inflammatory processes (59). It can inhibit the production of pro-inflammatory cytokines IL-1, IL-6, and IL-17, while also inducing the production of the anti-inflammatory cytokine IL-10 (60).

**Figure 1:**
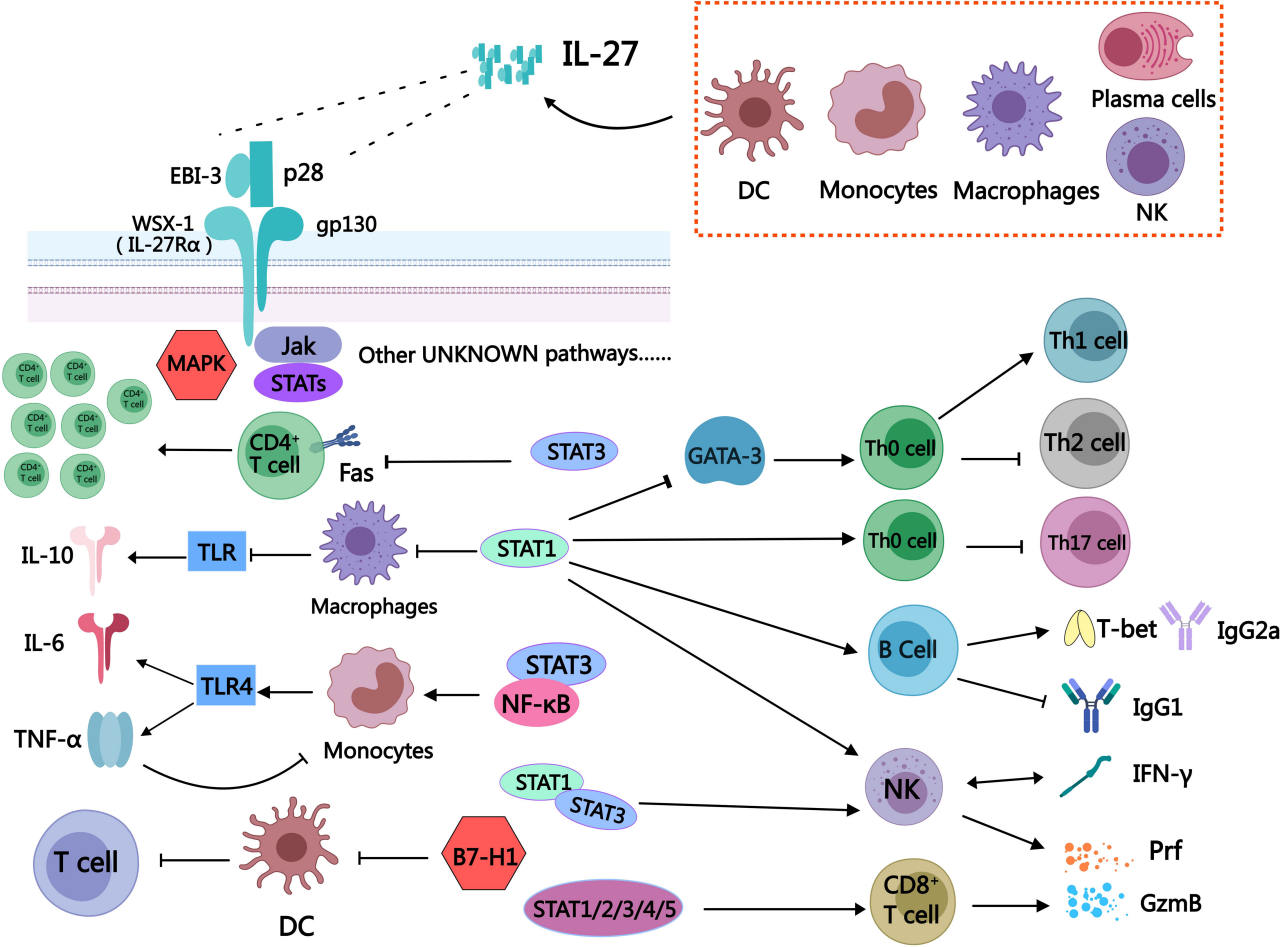
Schematic diagram of some immune cells regulated by IL-27. The secretion of IL-27 can be observed in various immune cells, including dendritic cells, monocytes, macrophages, plasma cells, and natural killer cells. IL-27 is a heterodimeric cytokine consisting of two subunits: EBI-3 and P28. The receptor for IL-27 is composed of WSX-1 (IL-27Rα) and gp130. Upon receptor activation, it exerts regulatory effects on downstream immune cells through JAK/STATs, MAPK pathways, as well as other signaling cascades that remain to be elucidated. IL-27 is a unique bidirectional regulator of inflammation, exerting a broad and significant regulatory role in diverse immune cell populations. It has the ability to activate STAT3, inhibit Fas-mediated apoptosis of CD4^+^T cells, and promote the expansion of CD4^+^T cells. Additionally, IL-27 facilitates the differentiation of Th0 cells into Th1 while suppressing their differentiation into Th2 by inhibiting GATA-3 expression through the STAT1 pathway. Moreover, IL-27 can impede the differentiation of Th0 cells into Th17 via activation of the STAT1 pathway. The stimulation of IL-27 can induce B cells to secrete T-bet and IgG2a, while inhibiting the secretion of IgG1 by B cells through STAT1 signaling. The expression of T-bet and GzmB in NK cells was promoted by IL-27 via the STAT1/3 pathway, thereby augmenting the functionality of NK cells through stimulation of IFN-γ secretion mediated by the STAT1 signal. The activation of STAT1/2/3/4/5 signaling in CD8^+^T cells by IL-27 leads to the direct release of Prf and GzmB, enabling the killing of tumor cells. The activation of STAT1 in macrophages by IL-27 leads to the augmentation of TLR-induced reduction in IL-10 and inflammation. Simultaneously, it attenuates the impact of TNF-α on human macrophages, thereby exerting an anti-inflammatory effect through a feedback mechanism. The expression of TLR4 in monocytes can be enhanced by IL-27 through the activation of STAT3/NF-κB signaling pathway, leading to the upregulation of downstream IL-6 and TNF-α expression. The expression of programmed death-related factor B7-H1 in DC can be up-regulated by IL-27, thereby inhibiting its function in promoting T cell proliferation.

The heterodimeric cytokine IL-27 is composed of Epstein-Barr virus-induced gene 3 (EBI3) and the p28 subunit of IL-12, which are linked by a disulfide bond (61), Specifically, the IL-12 cytokine family consists of four closely stacked alpha helices and long core spirochetes. The secretion of IL-27 is regulated by the immune system. In response to external stimuli, antigen-presenting cells (APCs) such as dendritic cells (DCs), monocytes, and macrophages can secrete IL-27. Additionally, a small amount of plasma cells, endothelial cells, and natural killer (NK) cells also have the capability to secrete IL-27. The Toll-like receptors (TLRs) pathway, serving as pattern recognition receptors within the evolutionarily conserved innate immune system, plays a pivotal role in initiating immune responses and bridging the gap between innate and adaptive immunity (62), the expression of IL-27 can be modulated by it (63) (**Figure 1**).

### 3.2 IL-27 receptor

The complex role of IL-27 may partly arise from its action on various cellular targets, with IL-27 signaling dependent on the membrane receptor IL-27R. IL-27R can be expressed on various immune cells, such as T cells, NK cells, B cells, monocytes, mast cells, dendritic cells, and endothelial cells (64). IL-27R is composed of WSX-1 (also known as IL-27Rα) and the gp130 subunit. WSX-1 is a transmembrane protein that acts as the α-chain of IL-27R and has a highly similar protein structure to gp130 (64). The gp130 subunit is expressed in immune tissues and cells, including the thymus, spleen, lymph nodes, and peripheral blood leukocytes, and plays a crucial role in immune regulation (65). When IL-27 exerts its effect, the p28 subunit binds to gp130 and EBI-3 associates with WSX-1, leading to receptor subunit heterodimerization and subsequent activation of downstream signaling pathways, the Janus kinase (JAK)/signal transducer and activator of transcription (STAT) as well as the MAPK signaling pathways are examples of such mechanisms (64) (**Figure 1**).

### 3.3 Cellular drivers and regulatory pathways of IL-27 expression

IL-27 is a heterodimeric cytokine composed of EBI3 and p28, encoded by genes located on chromosomes 19 and 16, respectively (66). The expression of IL-27 is regulated by immune stimuli, including IL-1β, TNF-α, and TLR ligands, which activate transcription factors such as NF-κB and IRF1 (67). This regulatory network ensures the precise transcription of EBI3 and IL27 genes, allowing IL-27 to respond to diverse inflammatory conditions and immune challenges (68). The primary sources of IL-27 are dendritic cells and macrophages, which produce IL-27 in response to specific environmental stimuli. For instance, TLR ligands such as LPS stimulate dendritic cells to release IL-27 and activate macrophages to upregulate its expression (69). These antigen-presenting cells tailor IL-27 production to the demands of the immune environment, ensuring its role in both initiating adaptive immunity and mitigating excessive inflammation.

IL-27 plays a dual role in immune regulation, balancing pro-inflammatory and anti-inflammatory responses. It promotes Th1-type immunity by inducing IFN-γ (70), while simultaneously limiting inflammation through the expansion of Tregs and the secretion of IL-10 (71). This bidirectional functionality allows IL-27 to maintain immune homeostasis across diverse pathological contexts, such as chronic inflammation, infections, and tumor microenvironments.

Understanding the structure and signaling pathways of IL-27 is essential for uncovering its potential effects on obesity-related metabolic diseases, particularly by examining its role in immune cells. Obesity is not only caused by an imbalance in energy intake and expenditure but also involves notable changes in the immune system and chronic inflammation. This chronic inflammation results from the activation and functional dysregulation of multiple immune cells in adipose tissue. IL-27, as a crucial immune regulatory factor, may significantly influence obesity-related inflammation and metabolic health through its effects on various immune cells.

## 4 Role of IL-27 in inflammatory immune cells

IL-27 plays a pivotal role in immune regulation by exhibiting both pro-inflammatory and anti-inflammatory effects, depending on the immune context and the specific cell types involved. As a cytokine that bridges innate and adaptive immunity, IL-27 exerts its functions on various immune cells, including T cells, B cells, macrophages, and dendritic cells. Its duality is critical for mounting effective immune responses against infections while preventing excessive inflammation that could lead to tissue damage or autoimmunity. To provide a clearer understanding, we have categorized IL-27’s effects into two sections: pro-inflammatory effects, where IL-27 enhances immune activation, and anti-inflammatory effects, where it acts to maintain immune tolerance and resolve inflammation.

### 4.1 Pro-inflammatory effects of IL-27

#### 4.1.1 Pro-inflammatory role of IL-27 in Th1 cells

IL-27 plays a significant pro-inflammatory role by promoting the differentiation and function of Th1 cells, which are primarily derived from naive CD4^+^ T cells (Th0). Th1 cells secrete IFN-γ, a key cytokine in cellular immunity. Studies have shown that in WSX-1 knockout mice, the Th1 immune response is impaired, IFN-γ production is significantly reduced, and the mice are prone to severe opportunistic infections (70). These findings highlight IL-27’s indispensable role in maintaining effective immune function (72). IL-27 facilitates Th1 differentiation through two main mechanisms: It prevents CD4^+^ T cell apoptosis via activation of the STAT3 signaling pathway (73). It alleviates the inhibitory effects of the transcription factor GATA-3 on Th1 differentiation (74) (**Table 1**).

**Table 1:** Role of IL-27 in Different Immune Cells.

Cell Type	Regulation Type	Specific Effects	Pathways	Mechanism	References
T Cells	Positive Regulation	Promotes Th1 cell differentiation, enhancing cell-mediated immune responses.	STAT3 GATA-3	Enhances Th1 differentiation through STAT3 signaling pathway and inhibition of GATA-3	(73, 74)
	Negative Regulation	Inhibits Th2 and Th17 cell differentiation, reducing excessive inflammation.	STAT1 GATA-3	Suppresses Th2 differentiation via STAT1 and inhibits Th17 development	(74, 79)
	Negative Regulation	Inhibits Th17 cell differentiation but has limited effects on fully differentiated Th17 cells.	STAT1	Suppresses Th0 differentiation into Th17 via STAT1 pathway	(79, 80)
	Positive Regulation	Activates CD8+ T cells, enhancing cytotoxic effects.	STAT1-5	Induces release of perforin and granzyme B, eliminating infected cells	(81)
B Cells	Positive Regulation	Promotes IgG2a secretion and enhances antibody production via STAT1 signaling pathway.	STAT1	Promotes IgG2a production and inhibits IgG1 secretion	(82, 91)
	Negative Regulation	Inhibits IgG1 secretion.	STAT1	Reduces IgG1 production, affecting antibody type distribution	(82)
NK Cells	Positive Regulation	Enhances NK cell function by upregulating T-bet and GzmB expression, stimulating IFN-γ secretion.	STAT1 STAT3	Upregulates T-bet and GzmB, enhances cytotoxicity and IFN-γ secretion	(83–85)
	Potential Regulation	In tumor microenvironment, may recruit and activate NK cells, potentially inhibiting tumor growth.	/	May influence NK cell function in tumors	(86)
DC Cells	Positive Regulation	Enhances DC migration and antiviral activity.	/	Improves DC antiviral capabilities	(89)
	Negative Regulation	Reduces antigen-presenting capacity and inhibits T cell proliferation by inducing B7-H1 expression.	/	Decreases antigen presentation and T cell proliferation	(92)
Mononuclear-Macrophages	Positive Regulation	Activates STAT3 and NF-κB pathways, increasing TLR4 factor expression, promoting pro-inflammatory factors like IL-6 and TNF-α.	STAT3 NF-κB	Enhances inflammatory response by increasing pro-inflammatory cytokines	(69)
	Negative Regulation	In human macrophages, IL-27 can have anti-inflammatory effects by reducing TNF-α and IL-1.	STAT1	Inhibits TNF-α and IL-1 production, reducing inflammation	(87, 92)

/ denotes “value missing or not applicable”.

These mechanisms underscore IL-27’s role in promoting Th1-mediated inflammation. However, excessive IL-27 activity can lead to tissue damage, requiring regulatory intervention. For example, in a rat model of arthritis, neutralizing antibodies against IL-27 significantly inhibited inflammatory factor production and alleviated arthritis symptoms (75).

#### 4.1.2 Pro-inflammatory role of IL-27 in Th2 cells

Th2 cells, derived from naive Th0, primarily exhibit anti-inflammatory, anti-allergic, and anti-rejection effects, contrasting with the pro-inflammatory roles of Th1 cells. However, IL-27 exerts a pro-inflammatory influence by inhibiting Th2 cell differentiation. It achieves this by directly suppressing the expression of the Th2-associated transcription factor GATA-3 via activation of the STAT1 pathway (74) (**Figure 1**).

In the absence of IL-27, the differentiation and activation of Th2 cells are significantly enhanced (76), which correlates with increased GATA-3 expression (77). By limiting Th2 cell generation, IL-27 prevents their anti-inflammatory effects, contributing to a shift toward pro-inflammatory immune responses.

#### 4.1.3 Pro-inflammatory role of IL-27 in Th17/CTL cells

Th17 cells, like Th1 and Th2, are derived from Th0. A unique feature of Th17 cells is their dual role in immune regulation, exhibiting both pro-inflammatory and anti-inflammatory effects. On one hand, Th17 cells help maintain intestinal integrity and reduce intestinal inflammation. On the other hand, they can disrupt lipid metabolism, contributing to obesity and associated metabolic disorders (78). IL-27 plays a regulatory role by inhibiting the differentiation of Th0 into Th17 cells through the STAT1 signaling pathway (79). However, IL-27 has minimal impact on fully differentiated Th17 cells (80) (**Table 1**). This selective inhibition suggests IL-27’s pro-inflammatory influence by shaping Th17 differentiation in a context-dependent manner.

Cytotoxic T lymphocytes (CTLs), a subset of CD8^+^ T cells, play a pivotal role in eliminating diseased cells. IL-27 enhances CTL activation via the STAT1/2/3/4/5 signaling pathways, promoting the release of perforin (Prf) and granzyme B (GzmB) (81) (**Table 1**). This pro-inflammatory effect strengthens the cytotoxic immune response, allowing IL-27 to facilitate the elimination of infected or abnormal cells while simultaneously contributing to inflammatory processes.

#### 4.1.4 Anti-inflammatory roles of IL-27 in B cells

IL-27 plays a significant pro-inflammatory role by regulating B cell class-switch recombination and the differentiation of specific subsets. Through the STAT1 signaling pathway, IL-27 rapidly activates primary splenic B cells in mice, inducing T-bet protein expression and promoting IgG2a class switching (82). IgG2a is a pro-inflammatory antibody that amplifies inflammatory responses through complement activation and enhanced phagocytosis (82). While IgG2a production is crucial for pathogen clearance, its overexpression may lead to uncontrolled inflammation, contributing to autoimmune diseases. Additionally, IL-27 suppresses IgG1 secretion, diminishing its anti-inflammatory effects and further promoting inflammation (82) (**Table 1**). These pro-inflammatory characteristics are essential in acute immune responses but may exacerbate tissue damage in chronic inflammatory diseases.

#### 4.1.5 Pro-inflammatory role of IL-27 in NK cells

NK cells play a vital role in identifying and eliminating viruses, infected cells, and tumors. IL-27 enhances NK cell activation and functionality, primarily through its interaction with IL-27 receptors, which exhibit high affinity on NK cells (83). IL-27 induces the upregulation of T-bet and GzmB expression via the STAT1 and STAT3 signaling pathways, thereby augmenting the cytotoxic capability of NK cells (84). Additionally, IL-27 stimulates NK cells to secrete IFN-γ through the STAT1 signaling pathway, further amplifying their immune activity (85).

In the tumor microenvironment, IL-27 promotes the recruitment and activation of NK cells, contributing to the suppression of tumor growth. Studies in mouse models have demonstrated that IL-27 effectively inhibits fibrosarcoma and melanoma by enhancing NK cell-mediated anti-tumor responses (86) (**Table 1**). These findings highlight IL-27’s pro-inflammatory role in boosting NK cell activity to combat infections and malignancies.

#### 4.1.6 Pro-inflammatory roles of IL-27 in mononuclear macrophage

IL-27 enhances the pro-inflammatory activity of the mononuclear-phagocyte system, which includes monocytes in the bloodstream and macrophages in tissues. In primary monocytes, IL-27 activates the STAT3 and NF-κB signaling pathways, increasing TLR4 expression and promoting the secretion of downstream pro-inflammatory cytokines such as IL-6 and TNF-α (69). In human macrophages, IL-27 stimulates the STAT1 pathway, reducing anti-inflammatory IL-10 levels and further amplifying the inflammatory response (87) (**Figure 1**; **Table 1**). These findings highlight IL-27’s role in driving inflammation, with its effects varying across species due to differences in IL-27 receptor expression.

#### 4.1.7 Pro-inflammatory roles of IL-27 in dendritic cells

IL-27 plays a pro-inflammatory role in DCs function by enhancing their migratory and antiviral capabilities. Research has demonstrated that IL-27 improves the migration of cord blood-derived dendritic cells and strengthens their antiviral responses, suggesting an important role in amplifying immune defenses (88). By boosting DCs activity, IL-27 contributes to the initiation and propagation of immune responses, highlighting its pro-inflammatory potential in various pathological contexts (**Figure 1**).

Despite IL-27’s prominent pro-inflammatory roles, such as enhancing the activity of T cells, B cells, and dendritic cells to promote immune responses, it also exhibits significant anti-inflammatory properties. This dual regulatory capability allows IL-27 to initiate immune defense while mitigating excessive inflammation when needed. Next, we will explore the anti-inflammatory effects of IL-27 and its critical mechanisms in maintaining immune homeostasis.

### 4.2 Anti-inflammatory effects of IL-27

#### 4.2.1 Anti-inflammatory effects of IL-27 in T cells

Obesity is often accompanied by chronic low-grade inflammation, where heightened activity of T cell subtypes like Th1 and Th17 exacerbates the inflammatory response (89). In this inflammatory context, IL-27, a key regulatory cytokine secreted primarily by dendritic cells, demonstrates notable anti-inflammatory effects. IL-27 can inhibit the differentiation of Th1 and Th17 cells by modulating STAT3 signaling pathways (39), thereby dampening pro-inflammatory responses. Moreover, IL-27 promotes the expansion and function of Tregs, which help restore immune balance and reduce inflammation (40). These mechanisms highlight IL-27’s critical role in mitigating inflammation and maintaining immune homeostasis in obesity-related conditions.

#### 4.2.2 Anti-inflammatory roles of IL-27 in B cells

Despite its prominent pro-inflammatory effects, IL-27 also exhibits anti-inflammatory properties under certain conditions. IL-27 regulates the balance of B cell subsets, preventing excessive inflammatory responses and maintaining immune homeostasis. For example, IL-27 selectively modulates B cell differentiation via the STAT1 and STAT3 signaling pathways, potentially inducing regulatory B cells that secrete anti-inflammatory cytokines such as IL-10 (90). Additionally, by enhancing the production of specific antibodies like IgG2a, IL-27 facilitates pathogen clearance, reducing persistent inflammatory stimuli and preventing chronic inflammation (82). These anti-inflammatory characteristics highlight IL-27’s critical role in balancing immune responses and protecting tissue integrity.

#### 4.2.3 Anti-Inflammatory mechanisms of IL-27 in dendritic cells

IL-27 exerts anti-inflammatory effects in DCs by inducing the expression of the programmed death factor B7-H1. This mechanism reduces the antigen-presenting capacity of DCs, leading to the inhibition of T lymphocyte proliferation (91). By limiting the activation and expansion of T cells, IL-27 helps regulate immune responses and prevents excessive inflammation. This highlights IL-27’s role in promoting immune homeostasis and mitigating immune overactivation in inflammatory conditions.

#### 4.2.4 Anti-Inflammatory roles of IL-27 in mononuclear macrophage

IL-27 exhibits notable anti-inflammatory effects in the mononuclear-phagocyte system, particularly in human macrophages. It suppresses the production of key pro-inflammatory cytokines, such as TNF-α and IL-1, by interfering with their signaling pathways (92) (**Table 1**). This suppression helps regulate macrophage activity, reducing excessive inflammation and promoting immune homeostasis. These findings emphasize IL-27’s role as a regulatory cytokine capable of mitigating inflammatory responses in various pathological conditions.

This dual functionality of IL-27, balancing pro-inflammatory and anti-inflammatory roles, underscores its importance in immune regulation. Such bidirectional effects become particularly significant in the context of chronic metabolic and inflammatory diseases, where IL-27’s role varies depending on the disease stage and microenvironment. In obesity and dyslipidemia, IL-27 promotes the activation of macrophages and Th1 cells, contributing to the chronic inflammation in adipose tissue and the progression of atherosclerosis (93). In type 1 and type 2 diabetes, IL-27 exhibits stage-dependent effects; it exacerbates pancreatic β-cell damage in early inflammation but later mitigates disease progression by suppressing Th17 responses and reducing antigen-presenting cell activity. In cardiovascular and cerebrovascular diseases, IL-27 acts as a double-edged sword, activating STAT3 pathways to reduce inflammation while also being implicated in pathological vascular remodeling. Additionally, IL-27’s influence on gut health through modulating microbial balance and intestinal barrier integrity highlights its systemic impact. These context-dependent actions demonstrate IL-27’s complexity in bridging immune regulation and metabolic health, necessitating a deeper understanding of its mechanisms across different diseases.

## 5 Role of IL-27 in obesity and other related metabolic diseases

### 5.1 IL-27 and obesity and dyslipidemia

**Figure 2:**
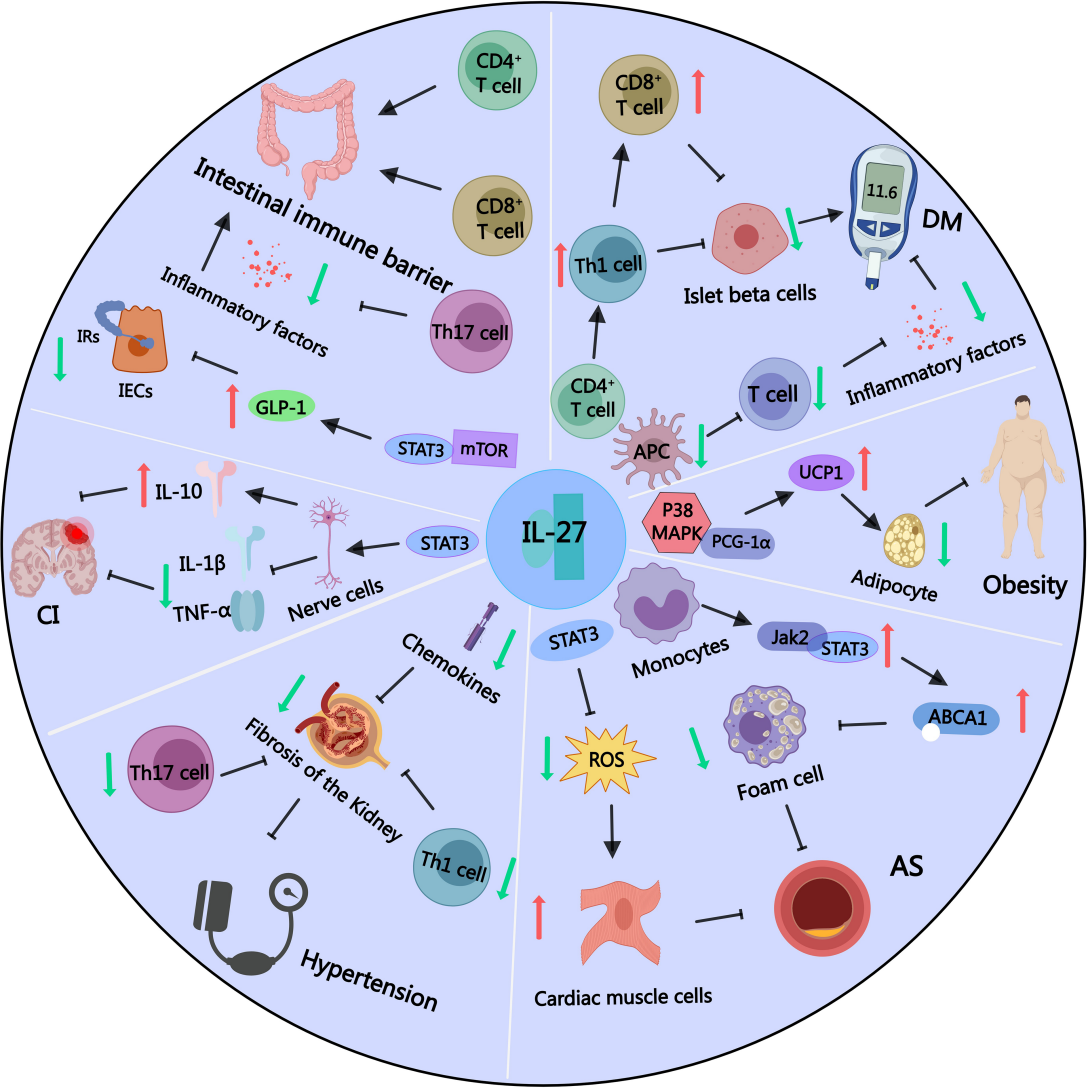
Part of IL-27 signaling pathways in obesity and other related metabolic diseases. IL-27 can promote the expression of thermogenic factor UCP1 by activating the p38MAPK-PGC-1α signaling pathway, depleting energy, reducing lipid accumulation, and inhibiting the occurrence of obesity. IL-27 can inhibit the activation of APC and T lymphocytes, reduce inflammatory factors, and inhibit the occurrence of diabetes. However, IL-27 can also affect the differentiation balance of CD4^+^ T lymphocytes, increase the maturation of Th1 pro-inflammatory cells, and promote the accumulation of CD8^+^ T lymphocytes, which can destroy islet β cells and promote the occurrence of diabetes. IL-27 can activate the JAK2/STAT3 signaling pathway in monocyte-macrophages, up-regulate the expression of ABCA1 protein, reduce the number of foam cells, and inhibit atherosclerosis. IL-27 can also activate STAT3 signaling pathway, reduce cellular reactive oxygen species level, reduce myocardial cell damage, and inhibit atherosclerosis. IL-27 can inhibit the excessive activation of CD4^+^ T lymphocytes, Th17 cells and chemokines, thereby inhibiting renal tubulointerstitial fibrosis and preventing the occurrence of secondary hypertension. IL-27 can activate STAT3 signaling pathway, reduce the levels of inflammatory molecules TNF-α and IL-1β, and increase the secretion of anti-inflammatory factor IL-10, thereby reducing the degree of nerve cell damage and the volume of cerebral infarction. IL-27 can activate STAT3-mTOR signaling, promote the synthesis and secretion of intestinal GLP-1, and alleviate IR. IL-27 can activate CD4^+^ and CD8^+^ cells in the intestinal intraepithelial lymphocyte population to provide an immune barrier to the intestine, and inhibit the differentiation of Th17 cells to reduce intestinal inflammation.

IL-27 has been identified as a key regulator of tissue thermogenesis, contributing to weight reduction and the inhibition of obesity. Clinical studies have shown that the expression level of the inflammatory cytokine IL-27 in the peripheral blood of obese individuals is significantly lower than that of normal-weight individuals (38). Interestingly, IL-27 levels increase following weight loss, suggesting a negative correlation between IL-27 and obesity in humans. Mechanistic studies reveal that IL-27 exerts its effects on thermogenesis independently of the traditional IL-27 receptor pathway. In Wsx-1 deficient mouse models, IL-27 directly activates the p38 MAPK-PGC-1α signaling axis, which is crucial for inducing thermogenic programs in brown and beige adipose tissue. This pathway enhances the expression of thermogenic genes, such as uncoupling protein 1 (Ucp1), and increases mitochondrial biogenesis, thereby promoting energy expenditure (38) (**Figure 2**). These findings highlight the non-canonical role of IL-27 in adipose tissue metabolism, establishing its potential as a therapeutic target for combating obesity.

Additionally, IL-27 mitigates chronic inflammation in white adipose tissue by regulating macrophage polarization from the pro-inflammatory M1 phenotype to the anti-inflammatory M2 phenotype, a classic pathway for reducing adipose tissue inflammation (93). These combined effects improve energy metabolism and prevent high-fat diet-induced obesity and related metabolic disorders. These findings underscore IL-27’s multifaceted role in regulating body weight through immune and metabolic pathways.

Clinical studies have observed a significant association between IL-27 single nucleotide polymorphism (SNP) and body mass index (BMI), suggesting that IL-27 may be inversely related to body weight. Specifically, genome-wide association studies (GWAS) have identified novel loci in IL-27-related pathways that correlate with BMI across multiple ethnic populations (94). This association implies that IL-27’s genetic variations might influence adipose tissue metabolism and inflammatory responses, contributing to its role in weight regulation. Supporting this, other studies indicate that reduced IL-27 levels are linked to hyperandrogenism, inflammation, and obesity-related conditions, such as polycystic ovary syndrome (PCOS) (95) (**Table 2**). These findings collectively suggest that IL-27 may play a protective role against excessive weight gain by modulating both systemic inflammation and adipose tissue function.

**Table 2:** Role of IL-27 in Obesity and Related Metabolic Diseases.

Disease/Condition	Role of IL-27	Specific Effects	Mechanism/Pathway	References
Obesity and Dyslipidemia	Promotes Thermogenesis	Enhances weight loss, inhibits obesity	p38 MAPK-PGC-1α signaling pathway	(38, 93–95)
	Anti-inflammatory	Reduces chronic inflammation in white adipose tissue	Involves STAT1 and STAT3	(93)
	Correlated with Weight	Lower IL-27 in obese individuals, increases after weight loss	/	(38, 94, 95)
T1DM	Pro-inflammatory	Promotes Th1 cell maturation, CD8+ T cell accumulation	Increases β-cell destruction	(16, 97, 98)
	Anti-inflammatory	Reduces immune infiltration, improves blood glucose levels	/	(100)
	Bidirectional regulation	Both pro- and anti-inflammatory effects	Involves STAT pathways	(96, 99, 100),,
T2DM	Varied Roles	Higher IL-27 in prediabetes, decreases in established T2DM	/	(103, 105, 106)
	Influences Complications	Linked to diabetic retinopathy	Correlates with glucose and lipid levels	(104)
	Inhibits T2DM Progression	Reduces APC and T-cell activity	Enhances inflammation	(105, 106)
Cardiovascular Disease	Pro-inflammatory in Atherosclerosis	Linked to coronary heart disease, increases with artery number	JAK2/STAT3 signaling pathway	(112, 114)
	Reduces Lipid Deposition	Increases cholesterol efflux, reduces foam cell formation	Induces ABCA1 protein expression	(114)
	Protects Cardiomyocytes	Reduces ROS, maintains calcium homeostasis	Involves STAT3	(115)
	Associated with Other CVDs	Elevated in ischemic heart disease, abdominal aortic aneurysm	Promotes inflammation	(113, 116–118)
Cerebrovascular Disease	Protective in Hemorrhage	Reduces edema, improves prognosis	Acts on neutrophils	(120)
	Protective in Ischemia-Reperfusion	Reduces neuronal stress injury, decreases infarction area	Activates STAT3, promotes IL-10 secretion	(121, 122)
Hypertension	Influences BP Fluctuations	Higher IL-27 in hypertensive patients	/	(124)
	Inhibits Renal Hypertension	Reduces renal fibrosis	Activates CD4^+^ T, Th17 cells	(20)
Gut Health	Reduces Insulin Resistance	Improves glucose and lipid metabolism	Activates STAT3-mTOR, promotes GLP-1 secretion	(126)
	Protects Gastrointestinal Epithelium	Decreases ulcers, increases cell proliferation	Upregulates STAT3 phosphorylation	(131)
	Maintains Gut Immune Function	Activates T lymphocytes, induces Treg cells	Prevents Th17 differentiation	(132, 133)
	Interacts with Gut Microbiota	Improves lipid levels and insulin resistance	Promotes IL-27 secretion by gut microbiota	(133)

/ denotes “value missing or not applicable”.

### 5.2 IL-27 and abnormal glucose metabolism

Diabetes is closely linked to obesity, which often drives the progression of glucose metabolism disorders by promoting insulin resistance and pancreatic inflammation. T1DM and T2DM are the two main types of diabetes, each with different pathogenesis. Research indicates that immune factors play a crucial role in the development and progression of both diseases. Specifically, the role of IL-27 in diabetes has garnered significant attention. Although its precise mechanism of action remains unclear, several key discoveries have been made.

#### 5.2.1 IL-27 and type 1 diabetes

Research indicates that CD4^+^ T cells and CD8^+^ T cells work together to drive the destruction of pancreatic β-cells in T1DM, with CD8^+^ T cells playing a predominant pro-inflammatory role (16). Meta-analyses have revealed associations between T1DM and cytokines such as IL-27, IL-10, IL-19, and IL-20, suggesting that IL-27 may be a key immunomodulator in T1DM (96). In non-obese diabetic mouse models, IL-27 promotes the differentiation and activation of Th1 cells via the STAT1 pathway and drives the accumulation of pro-inflammatory CD8^+^ T cells in pancreatic islets, exacerbating β-cell destruction and inflammation (97, 98) (**Figure 2**). Antibody-based neutralization of the IL-27 subunit p28 has been explored as a therapeutic strategy, effectively blocking its activity to reduce immune-mediated β-cell damage (99).

Conversely, IL-27 has shown anti-inflammatory properties in specific contexts of T1DM. In streptozotocin-induced diabetic mouse models, knockout of the IL-27 subunit EBI3 or its receptor complex results in increased immune cell infiltration into pancreatic islets and aggravated hyperglycemia. IL-27 administration in these models reduces immune infiltration and restores blood glucose levels, possibly by promoting Treg expansion and enhancing IL-10 production (100) (**Table 2**). These findings suggest that IL-27’s role in T1DM may depend on the immune context and disease stage, demonstrating both pro-inflammatory and anti-inflammatory characteristics.

Clinical studies on the relationship between IL-27 and T1DM remain limited. While one study found no association between IL-27 gene variants and T1DM susceptibility in a South American population (101), its small sample size and geographic limitations leave this conclusion uncertain. Taken together, these findings highlight the dual roles of IL-27 in T1DM, with its pro-inflammatory effects exacerbating β-cell destruction and its anti-inflammatory properties potentially mitigating disease progression. Future research should focus on clarifying the context-dependent mechanisms of IL-27 in T1DM, which will be critical for optimizing its therapeutic targeting.

#### 5.2.2 IL-27 and type 2 diabetes

The pathogenesis of T2DM is multifaceted, involving insulin resistance, β-cell dysfunction, obesity, and genetic factors. Inflammation plays a pivotal role in these processes, directly driving insulin resistance and β-cell damage, and indirectly disrupting glucose metabolism through changes in the metabolic environment. Clinical studies demonstrate that individuals with dyslipidemia, often accompanied by chronic inflammation, are at a higher risk of developing T2DM (17). Additionally, systemic inflammation during hyperglycemia exacerbates lipid metabolism disturbances, further worsening glucose homeostasis (102).

The role of IL-27 in T2DM appears to be complex and stage-dependent. Elevated levels of IL-27 in peripheral blood have been observed in prediabetic and newly diagnosed diabetic patients, suggesting that IL-27 might promote the progression of early T2DM by enhancing inflammation and immune cell activation (103). Mechanistically, IL-27 can activate APCs and stimulate Th1 cell differentiation through the STAT1 pathway, thereby amplifying pro-inflammatory responses. Clinical studies further reveal that IL-27 is associated with T2DM complications, such as diabetic retinopathy, where IL-27 concentrations in aqueous humor positively correlate with blood glucose, lipid levels, and HbA1c (104).

Conversely, reduced IL-27 levels in advanced T2DM have been linked to worsened disease progression. Mechanistic studies suggest that a lack of IL-27 secretion reduces its potential anti-inflammatory effects, such as suppressing APCs activity and promoting Tregs expansion (105) (**Figure 2**). This deficiency may result in heightened systemic inflammation and immune dysregulation, exacerbating β-cell damage and insulin resistance. Genetic studies support this dual role of IL-27, with polymorphisms in the IL-27 gene being associated with insulin resistance and subclinical atherosclerosis in T2DM (106) (**Table 2**). Together, these findings suggest that IL-27 exerts both pro-inflammatory and anti-inflammatory effects in T2DM, depending on the stage of the disease and immune context, highlighting the need for further research to clarify its therapeutic potential.

### 5.3 IL-27 and cardiovascular disease

Obesity triggers chronic low-grade inflammation, heightening the risk of cardiovascular diseases. Inflammatory factors directly damage the cardiovascular system and contribute to cardiovascular disease by promoting atherosclerosis and endothelial dysfunction (18). Consequently, obesity is regarded as a major risk factor for cardiovascular diseases. Mechanistically, the inflammatory response in cardiovascular diseases is associated with the imbalance of anti-inflammatory and pro-inflammatory factors, involving T lymphocytes, macrophages, neutrophils, and related cytokines (107). Notably, CD4^+^ T lymphocyte subsets are closely linked to atherosclerosis and coronary artery disease (108).

Atherosclerosis development is driven by pro-inflammatory cytokines and Th1 cells, whereas anti-inflammatory cytokines and Tregs counteract this process. IL-27, a cytokine that modulates nearly all T cell subsets, orchestrates the immune response underlying cardiovascular pathology by balancing these pro-inflammatory and anti-inflammatory pathways (60, 109). Genetic mutations in the IL-27 p28 subunit enhance its inflammatory potential, as seen in early-onset atherosclerosis and Kawasaki disease patients with the rs17855750 variant, which predisposes individuals to coronary artery disease (110, 111).

Mechanistically, IL-27 amplifies the pro-inflammatory cascade in coronary artery disease by increasing LDL-induced dendritic cell activation, which stimulates the release of inflammatory mediators that promote vascular injury (112). Elevated IL-27 levels correlate with greater coronary artery stenosis, driven by enhanced Th1 polarization and cytokine secretion, directly contributing to the recruitment of monocytes and neutrophils at lesion sites. In abdominal aortic aneurysms, IL-27 receptor knockout reduces inflammatory infiltration and delays disease onset, underscoring its role in exacerbating vascular inflammation (113).

Conversely, IL-27 mitigates lipid accumulation in atherosclerotic plaques via the JAK2/STAT3 signaling pathway. In macrophages, IL-27 upregulates ABCA1 expression, enhancing cholesterol efflux and reducing foam cell formation, thereby attenuating plaque progression (114). In cardiomyocytes, IL-27 protects against oxidative damage and calcium overload through STAT3 activation, maintaining cellular homeostasis in high-glucose and high-cholesterol environments (115). Moreover, IL-27’s duality extends to ischemic heart disease, where elevated serum levels enhance immune cell activation during acute myocardial infarction while simultaneously reducing oxidative stress and stabilizing atherosclerotic plaques through STAT3 signaling (116) (**Table 2**; **Figure 2**).

Beyond coronary and vascular diseases, IL-27 genetic polymorphisms influence cardiac conditions, such as dilated cardiomyopathy and atrial fibrillation. These polymorphisms may disrupt STAT3-mediated protective signaling, altering calcium homeostasis and increasing arrhythmogenic potential (117, 118) (**Table 2**). Although further research is needed to elucidate these pathways, the evidence underscores IL-27’s context-dependent effects, as it mediates both pro-inflammatory damage and anti-inflammatory repair processes in cardiovascular diseases.

### 5.4 IL-27 and cerebrovascular diseases

Similar to cardiovascular disease, inflammation caused by cerebrovascular disease is the main pathological basis of atherosclerosis (19). Obesity as a result of sustained inflammatory state will increase the risk of cerebrovascular diseases such as cerebral infarction (119). Long-term chronic inflammation increases vascular fragility, making it prone to rupture and bleeding when blood pressure rises.

In this context, the role of IL-27 in cerebrovascular diseases has gained increasing attention. Research has found that IL-27 in peripheral blood rapidly increases in the early stages of cerebral hemorrhage, exerting a protective role. IL-27 acts on neutrophils through its receptor, inducing the release of protective factors like lactoferrin and haptoglobin, while inhibiting the activity of inflammatory molecules, effectively reducing edema and improving prognosis (120). During cerebral ischemia-reperfusion, IL-27 can reduce neuronal stress injury and decrease the area of cerebral infarction. Mechanistic studies indicate that IL-27 activates the STAT3 signaling pathway, promoting the secretion of the anti-inflammatory cytokine IL-10 while reducing the secretion of pro-inflammatory cytokines TNF-α and IL-1β (121) (**Figure 2**). A clinical study observed significant differences in 10 cytokines, including IL-27, in the serum of acute and chronic cerebral infarction patients through chip detection and serum screening, but the related mechanisms were not further investigated (122) (**Table 2**).

### 5.5 IL-27 and hypertension

Obesity through multiple mechanisms, including inflammation, endocrine factors change, metabolic disorders, mechanical pressure increase, and sleep apnea, directly or indirectly lead to high blood pressure in the occurrence and development. These mechanisms, has led to obesity significantly increased the risk of high blood pressure, and obesity related hypertension is usually difficult to control (123).

IL-27 may exert a certain influence on blood pressure fluctuations. In a cross-sectional study, the levels of IL-27 were found to be higher in hypertensive patients compared to the control group, and there was a positive correlation between the degree of blood pressure elevation and IL-27 (124). However, whether IL-27 plays an inhibitory or promotive role in hypertension requires further investigation.

In the etiology of secondary hypertension, the kidney holds significant importance, with inflammatory factors playing a crucial regulatory role (125). Mechanistically, IL-27 appears to inhibit renal fibrosis, a key factor in the development of renal hypertension. Experimental studies show that IL-27’s protective effects are mediated through its regulation of CD4^+^ T lymphocytes and Th17 cells, which suppress fibrotic processes in renal tissue. Loss of IL-27 signaling, as observed in IL-27Rα-deficient models, exacerbates tubulointerstitial fibrosis and enhances Th17-mediated inflammatory responses, highlighting its potential inhibitory role in renal hypertension (20) (**Figure 2**).

### 5.6 IL-27 and the gut

Recent research indicates that obesity is not only caused by an imbalance between energy intake and expenditure but is also closely related to chronic low-grade inflammation. The gut is the body’s largest immune organ, and its health has a significant impact on overall metabolism and inflammatory responses. Obesity is often accompanied by intestinal inflammatory responses, including weakened intestinal mucosal barrier function and dysbiosis of the gut microbiota, which further exacerbate obesity and its complications (21).

#### 5.6.1 IL-27 and glucagon-like peptide-1

Mechanistically, IL-27 activates the STAT3-mTOR signaling pathway in obese mice, promoting the synthesis and secretion of GLP-1 in the gut, thereby reducing insulin resistance and alleviating glucose and lipid metabolism disorders associated with obesity (126) (**Table 2**; **Figure 2**). However, studies investigating the connection between IL-27 and GLP-1 remain limited, offering only a narrow understanding of this relationship. Interestingly, IL-27 shares significant similarities with other interleukins, such as IL-6 and IL-10, in terms of receptor composition and signaling pathways. For example, the IL-27 receptor is composed of gp130 and WSX-1 subunits, with gp130 being a shared component of the IL-6 cytokine family, suggesting potential functional overlaps in their signaling pathways. Additionally, both IL-27 and IL-10 depend on STAT3 for downstream gene regulation, though their target cells and functions differ. IL-10 predominantly exhibits anti-inflammatory and immunosuppressive effects, while IL-27 plays dual roles in inflammation and immune modulation. Despite this, the potential link between IL-27 and GLP-1 in mucosal immunity remains underexplored, but studies on IL-6 and IL-10 provide valuable insights.

Among other interleukins, IL-6 has been extensively studied for its role in regulating GLP-1 secretion and related metabolic effects. IL-6 promotes GLP-1 secretion from intestinal L cells and pancreatic alpha cells via the JAK/STAT3 signaling pathway, improving insulin secretion and glucose tolerance (127). Additionally, IL-6 signaling in adipocytes induces intestinal GLP-1 secretion, suggesting a broader role of metabolic cytokines in systemic energy balance (128). However, some studies indicate that acute IL-6 administration does not significantly increase GLP-1 secretion, which may depend on the timing and tissue specificity of inflammatory stimuli (129). IL-10 has also been shown to mediate GLP-1 receptor-induced anti-inflammatory and analgesic effects via β-endorphin expression in spinal microglia (130). These mechanisms suggest that IL-27 might share similar functions with IL-6 and IL-10, but further experimental studies are needed for confirmation. Current evidence indicates a potential role for IL-27 in regulating GLP-1 secretion in the gut, though the underlying mechanisms remain unclear. Building on the findings from IL-6 and IL-10 studies, future research should explore whether IL-27 activates gut immune and endocrine pathways to promote GLP-1 secretion, thereby improving insulin resistance and alleviating glucose and lipid metabolism disorders associated with obesity. This could provide a theoretical basis for developing metabolic interventions targeting IL-27.

#### 5.6.2 IL-27, gut epithelium, and microbiota

Additionally, IL-27 protects gastrointestinal epithelial cells by reducing epithelial ulcers and enhancing the proliferation rate of damaged cells. This protective effect is mediated by the upregulation and phosphorylation of STAT3 expression stimulated by IL-27 (131). IL-27 derived from intestinal epithelial cells plays a dual role in gut immunity. It specifically activates CD4^+^ and CD8^+^ T lymphocytes within the epithelium, forming an immune barrier for the gut, while IL-27 secreted by innate immune cells induces Treg cell differentiation to prevent immune overactivation (132) (**Figure 2**). During intestinal inflammation, Treg cells can produce IL-27, limiting the differentiation of pro-inflammatory Th17 cells and alleviating intestinal inflammation (133) (**Table 2**). These coordinated actions of IL-27 from various sources collectively maintain normal gut immune function, reduce insulin resistance, and inhibit the progression of obesity.

Emerging evidence highlights a potential link between IL-27 and gut microbiota. A study investigating the effects of Angelica keiskei Jiaosu, prepared through yeast fermentation, found that this fermentation product upregulated IL-27 levels in the serum of high-fat diet-fed mice (134). This increase in IL-27 was associated with improvements in blood lipid profiles and a reduction in insulin resistance, suggesting a possible role of IL-27 in mediating the anti-obesity effects of gut microbiota-modulating interventions, but further mechanistic investigations are needed to clarify the interactions between IL-27 and microbiota.

#### 5.6.3 IL-27 and inflammatory bowel disease

Building on IL-27’s critical role in gut immunity, its regulatory mechanisms in chronic inflammatory diseases such as inflammatory bowel disease (IBD) have garnered significant attention. Studies have shown that elevated IL-27 levels are closely associated with disease activity in Crohn’s disease (CD). In inflamed intestinal tissues of CD patients, IL-27 expression is significantly upregulated and positively correlated with disease severity (135). IL-27 acts as a key immune regulator in CD, particularly by influencing antigen presentation and processing in intestinal epithelial cells. It has been identified as a novel regulator of major histocompatibility complex (MHC) class I and class II expression, enhancing the interaction between epithelial cells and T cells, thereby shaping immune responses (136). These findings indicate that IL-27 plays a pivotal role not only in orchestrating immune responses but also in facilitating communication between epithelial and immune cells.

Genetic studies have further elucidated the role of IL-27 in CD. Polymorphisms in the IL-27 gene, such as rs153109, have been significantly associated with increased susceptibility to CD in the Chinese Han population, suggesting that IL-27 may influence disease predisposition at the genetic level (137). Functionally, IL-27 interacts with other immune regulatory pathways. For example, the gut commensal bacterium Faecalibacterium prausnitzii promotes an anti-inflammatory phenotype in DCs by inducing IL-27, IL-10, and other immunoregulatory molecules, thereby promoting the differentiation of IL-10-secreting T cells (138). Conversely, in experimental IBD models, deficiency of the IL-27R delays the onset of colitis and reduces inflammation in chronic IBD, highlighting the context-dependent and dual roles of IL-27 in CD pathogenesis (139).

The role of IL-27 in ulcerative colitis (UC) also demonstrates dual functionality, exhibiting both anti-inflammatory and pro-inflammatory effects depending on the context. Genetic studies reveal that specific IL-27 polymorphisms may exert varying effects in different individuals. One study found that polymorphisms in the IL-27 gene, such as rs153109, confer a protective effect against UC, potentially by modulating immune responses and reducing inflammation severity (140). Conversely, another study in a northern Chinese Han population identified a functional variant in the promoter region of the IL-27 gene that enhances its transcriptional activity and increases susceptibility to UC, suggesting that IL-27 may exacerbate disease under certain conditions (141). These findings indicate that while IL-27 has protective, anti-inflammatory properties in UC, it may also exhibit pro-inflammatory potential influenced by genetic and environmental factors.

The role of IL-27 in IBD differs by disease subtype. In Crohn’s disease, IL-27 tends to exert pro-inflammatory effects by promoting Th1 differentiation and IFN-γ secretion, though it also has regulatory functions such as inducing IL-10 production. In ulcerative colitis, IL-27 demonstrates protective and anti-inflammatory characteristics, though its effects may be influenced by genetic and environmental factors. These findings underscore IL-27’s dual regulatory role and highlight its importance in the pathophysiology of IBD.

## 6 Potential and challenges of IL-27 as a target for the treatment of metabolic diseases such as obesity

Obesity and its related metabolic diseases are usually accompanied by chronic low-grade inflammation. This chronic inflammation not only affects adipose tissue but also involves inflammatory responses in multiple organs throughout the body, such as the pancreas, liver, blood vessels, and intestines. Inflammatory pathways associated with obesity and metabolic diseases are promising therapeutic targets. Studies have shown that traditional inflammatory factors such as TNF-α play important roles in obesity-related insulin resistance. However, it has been observed that the use of anti-TNF-α antibodies alone does not effectively improve insulin resistance (142). This suggests that in inflammation treatment, targeting a single cytokine, chemokine, or pathway may not produce the desired effects. Multi-target regulatory molecules represented by IL-27 have become a new research direction. The cytokine IL-27 has a bidirectional regulatory role in inflammation control. For example, in adipocytes, IL-27 can act as an immune activator or regulator, controlling the complex metabolic functions of adipocytes and influencing the progression of obesity-related diseases (143). The different mechanisms of action of IL-27 in different types of cells make it a potentially valuable tool in treating obesity and its related metabolic diseases.

### 6.1 The role of IL-27 in adipose tissue

IL-27 molecules have various sources, which may lead to different functions depending on their origin (132). The secretion of IL-27 molecules primarily comes from DCs and macrophages (144), but adipocytes also secrete IL-27 (143). Recently, it has been discovered that IL-27 secreted by CX3CR1^+^ cells from sympathetic neuron-associated macrophages specifically targets adipocytes, enhancing thermogenesis and energy expenditure, indicating that IL-27 plays an important role in regulating energy metabolism (38). Additionally, there may be a synergistic regulatory mechanism between sympathetic neuron macrophages and norepinephrine in thermogenesis (145), further enhancing the therapeutic effect of IL-27 on obesity. The specific targeting of adipocytes by IL-27 secreted by CX3CR1^+^ cells warrants further investigation (146). Based on previous discussions, we speculate whether IL-27 from different cell sources has conformational or modification differences, leading to different biological effects. With advances in modern computational and bioengineering technologies, is it possible to design cytokines with different affinities for specific cell receptors, or customize biased IL-27 variants to enhance the regulation of energy metabolism while reducing potential toxic side effects?

### 6.2 Bidirectional regulatory role of IL-27

IL-27’s unique bidirectional regulatory role enables it to both inhibit excessive inflammatory responses and activate inflammatory molecules when appropriate to exert biological effects. This bidirectional regulation primarily relies on the biological structure of IL-27 and its specific receptors. The two subunits of IL-27, EBI-3 and p28, have different biological functions. EBI-3 is typically a promoter of inflammatory initiation. Notably, p28 can independently inhibit IL-6-mediated Th17 cell activation, exerting anti-inflammatory effects (147). The p28/CLF protein can induce STAT1/3 phosphorylation, inhibiting the proliferation of CD4^+^ T lymphocytes, thereby also playing an anti-inflammatory role (148).

Furthermore, the bidirectional role of IL-27 in inflammation is closely related to its receptors. When IL-27 acts on cells, the EBI-3 subunit binds to the receptor WSX-1, and the p28 subunit binds to the receptor gp130. IL-27 receptors WSX-1 and gp130 co-express and form a dimer, initiating downstream signaling pathways (64). WSX-1 is particularly unique, as IL-27 is the only known ligand for this orphan receptor. Previously, WSX-1 was observed to be highly expressed in T lymphocytes, B cells, and macrophages (149), leading to research focusing primarily on immune cells. However, the recent discovery of IL-27R receptor WSX-1 in non-immune cells (adipocytes) provides a new therapeutic target for obesity-related metabolic disorders. Additionally, this finding suggests that IL-27R may be expressed in other non-immune cells in the body (38).

Moreover, the two subunits of the IL-27 receptor are not consistently expressed during T lymphocyte activation, with WSX-1 upregulated and gp130 downregulated, possibly leading to differences in their affinity for IL-27 subunits and thus different immunoregulatory effects. Given the relatively recent discovery of IL-27, its structural exploration has not been deeply conducted. However, insights can be drawn from the structurally similar IL-2. IL-2 receptors are classified into high, medium, and low affinity categories. The high-affinity receptor is a trimer composed of alpha, beta, and gamma subunits, while the medium-affinity receptor is a dimer composed of beta and gamma subunits. IL-2 preferentially binds to high-affinity receptors at low concentrations, promoting Treg cell proliferation and inhibiting inflammatory responses and excessive stress. At higher concentrations, IL-2 preferentially binds to medium-affinity receptors, promoting CD8^+^ T lymphocyte and NK cell proliferation, enhancing immune responses (150). This mechanism suggests that IL-27 may also have similar unknown molecular switches or alternative receptors, with functions potentially influenced by intervention concentrations and other factors. In-depth research into these mechanisms will help better understand IL-27’s role in immune regulation.

### 6.3 Potential and challenges of clinical applications

According to our search on clinicalTrials.gov, clinical trials on IL-27 therapy are limited, mainly focusing on highly malignant tumors such as hepatocellular carcinoma (151), refractory/advanced solid tumors (152), recurrent or progressive central nervous system tumors, and recurrent or metastatic nasopharyngeal carcinoma (153). Exogenous IL-27 antibodies act as immune checkpoints, promoting the reactivation of cells like NK cells through binding with IL-27, thus exerting strong anti-tumor effects (151). The fully human anti-IL-27 antibody SRF388, currently in phase II clinical trials, has been granted orphan drug status by the FDA. It effectively inhibits the interaction between IL-27 and its receptor WSX-1, blocking downstream signals of the JAK-STAT pathway, enhancing the activation of NK cells and immune cells, thereby slowing the progression of hepatocellular carcinoma (151). However, the role of IL-27 in obesity-related metabolic diseases requires further clinical trial research.

Determining the appropriate intervention concentration is a major challenge in drug development, as it may sometimes cause severe side effects such as cytokine storms (154). Interleukin preparations usually have a short half-life and high toxicity at high doses (155). When IL-2 and IL-7 are combined with CAR-T therapy, as the number of CAR-T cells increases, the secretion of IFN-γ and TNF-α also increases correspondingly (156), potentially triggering a cytokine storm (157). Therefore, it is necessary to carefully balance the benefits and risks of dosage. To achieve therapeutic effects, it is essential to explore optimized drug delivery methods and administration strategies, such as antibody and cytokine fusion proteins, molecular targeting methods, etc., to improve the efficacy of cytokines and reduce side effects (158). Intraperitoneal injection of IL-27 can reduce weight in wild-type obese mice, improve insulin resistance and fatty liver without causing systemic inflammation or tissue damage (38). These potential mechanisms are worth further investigation.

Despite significant progress in cytokine animal experiments, substantial differences remain in actual clinical applications. The genetic similarity of mice is often considered to better reflect human biology, but differences in innate immunity, adaptive immunity, activation, and response to external stress are often overlooked. The proportion of neutrophils in human peripheral blood is 50% to 70%, while in mice it is only 10% to 25%. Additionally, the proportion of lymphocytes and the expression of surface molecules on macrophages differ (159). These differences may lead to success in mouse experiments but failure in human clinical trials. Recently, the highly anticipated anti-IL-23 p19 monoclonal antibody Risankizumab failed to show significant efficacy in a phase IIa clinical trial for adult severe asthma (160).

### 6.4 Future research directions

To apply IL-27 in the clinical treatment of obesity and related metabolic diseases, future research should focus on several aspects:1) Investigate the mechanisms of IL-27 in different tissues and cell types, particularly its metabolic regulatory function in adipocytes. Attention should be given to the issue of target specificity, ensuring that the drug acts on diseased tissues without affecting healthy ones, which is a challenge in drug development. 2) Optimize the delivery methods and formulations of IL-27 to enhance its stability and effective concentration *in vivo*. The development of personalized precision medicine, localized delivery, and combined therapy with existing methods should also be prioritized. 3) Develop animal models that better mimic human immune function to improve the clinical translatability of research findings. 4) The interaction of IL-27 with other cytokines and signaling pathways is complex. It is crucial to explore these interactions and design more effective multi-target therapeutic strategies. 5) In clinical trials, stratified research on different patient groups (such as obese and diabetic patients) can provide data to support large-scale applications in the future. The long-term effects and safety of IL-27-targeted therapies, especially in chronic disease patients, should be evaluated. This includes assessing whether prolonged use of IL-27 could lead to immune dysregulation or other side effects, such as an increased risk of infections or the development of autoimmune diseases.

In conclusion, IL-27, as a multifunctional cytokine, shows broad application prospects in the treatment of obesity and its related metabolic diseases. However, its clinical application still faces many challenges and requires further research and optimization. Through continuous exploration and innovation, IL-27 is expected to become an effective target for addressing obesity-related metabolic diseases.
